# Schizophrenia and other psychotic disorders in Caribbean-born migrants and their descendants in England: systematic review and meta-analysis of incidence rates, 1950–2013

**DOI:** 10.1007/s00127-015-1021-6

**Published:** 2015-02-07

**Authors:** A. Tortelli, A. Errazuriz, T. Croudace, C. Morgan, R. M. Murray, P. B. Jones, A. Szoke, J. B. Kirkbride

**Affiliations:** 1 INSERM, U955, Equipe 15, Créteil, 94000 France; 2 Department of Psychiatry, University of Cambridge, Cambridge, UK; 3 Department of Nursing and Midwifery, University of Dundee, Dundee, UK; 4 Institute of Psychiatry, King’s College London, London, UK; 5 AP-HP, Groupe Hospitalier “Mondor”, Pôle de Psychiatrie, INSERM, Créteil, 94000 France; 6 Division of Psychiatry, UCL, Charles Bell House, 67-73 Riding House Street, London, W1W 7EJ UK

**Keywords:** Psychotic disorders, Ethnicity, Migration, Systematic review, Incidence, Meta-analysis

## Abstract

**Purpose:**

Increased risk of schizophrenia and other psychotic disorders among black Caribbean migrants and their descendants have been described since the 1960s. It remains unclear whether this risk varies over time, between rural and urban areas, or according to methodological artefact.

**Methods:**

We conducted a systematic review of the incidence of adult-onset psychotic disorders in black Caribbean groups relative to the baseline population in England, published 1950–2013. Subject to sufficient data (*N* ≥ 5) we used random effects meta-analyses to estimate pooled incidence rates (IR) and rate ratios (IRR) of seven psychotic disorder outcomes, and meta-regression to inspect whether any variation was attributable to study-level methodological features, including case ascertainment, denominator reliability, choice of baseline population and study quality.

**Results:**

Eighteen studies met inclusion for review. Sixteen demonstrated statistically significant elevated incidence rates in the black Caribbean group, present across all major psychotic disorders, including schizophrenia and bipolar disorder. Methodological quality increased over time (*p* = 0.01), but was not associated with estimated IR or IRR. For schizophrenia (*N* = 11 studies) the pooled IRR in the black Caribbean group was 4.7 (95 % CI 3.9–5.7) relative to the baseline; no evidence of publication bias was observed. We found weak evidence to suggest schizophrenia IRRs were smaller from studies in more urban settings (odds ratio 0.98; 95 % CI 0.96–1.00; *p* = 0.06).

**Conclusions:**

Higher incidence rates of psychotic disorders have been present for more than 60 years amongst black Caribbean ethnic groups in England, despite improved study methodologies over time. Aetiological explanations appear to more parsimoniously account for this excess than methodological biases.

**Electronic supplementary material:**

The online version of this article (doi:10.1007/s00127-015-1021-6) contains supplementary material, which is available to authorized users.

## Introduction

The risk of developing schizophrenia and other psychotic disorders varies by both migrant and minority ethnic status [1, 2]. This finding is far from new [3], and has been observed on four separate continents [4–8]. Meta-analyses suggest that the risk of schizophrenia for first and second generation migrants is between 2 and 4.5 times that of the majority ethnic group under study [1, 9]. Of course, these figures belie heterogeneity in risk, with excess rates reported in individual studies ranging 50–1,400 % (i.e. [4, 10]).

This heterogeneity may be aetiological or artefactual in nature. Aetiological hypotheses put forward to explain the excess risk observed in some minority ethnic groups centre around the identification of other risk factors (i.e., age, sex, socioeconomic status, discrimination, social isolation, genetic factors, infections, stress, substance misuse) [11], which could potentially confound or otherwise account for the relationship between psychosis and minority ethnic position (i.e., the potential role of neighbourhood ethnic density [12]). All other explanations for the excess rates of psychotic disorder in minority ethnic groups must arise from an artefact (i.e., defect) of observation (including reverse causation [13, 14]). In incidence-based studies, these biases may affect accurate ascertainment of the numerator (i.e., cases) or denominator (i.e., the population at-risk), and may have differential or non-differential effects on estimating rates of psychotic disorders by ethnicity. Epidemiological research seeks to minimise the latter (artefactual) explanations, while maximising the opportunity to detect confounders, effect modifiers and other factors on the causal pathway between psychosis risk and minority ethnic status.

The earliest studies of raised rates of psychotic disorder amongst labour immigrants in the UK paid little heed to either set of explanations. Instead, they provided observational evidence of a higher prevalence of consultation rates for schizophrenia among people born in the Caribbean or West Africa, compared with their British-born counterparts [15, 16]. Subsequent studies in the 1960s made some methodological improvements over this early research, restricting comparisons to first hospitalised admissions, finding an excess of clinically diagnosed schizophrenia in Caribbean communities in Southeast London [17] and Nottingham [18]. The interpretation of these early reports was limited by both aetiological and artefactual issues, including a lack of population-based case finding, the absence of operationalised and standardised diagnostic criteria for a range of psychotic disorders (not limited to schizophrenia), imprecise estimates of the underlying population at-risk and a lack of control for basic confounders, such as age and sex. Cochrane [19] conducted the first study of psychiatric admission rates by country of birth to employ age-sex standardisation, using population data from the 1971 Census, marking a significant improvement in methodological quality; first generation Caribbean-born migrants remained nearly four times more likely to be admitted for schizophrenia and related disorders (21.5 per 100,000 person-years) than the English-born population (5.8 per 100,000 person-years).

Subsequent studies have continued to observe elevated rates of schizophrenia and other psychotic disorders in minority ethnic groups [1, 9], against a backdrop of attempts to improve methodological rigour. Both aetiological and artefactual explanations have come into research focus. For example, in the UK, Kirkbride et al. [20] have shown that elevated rates of psychotic disorder were attenuated by up to 40 % in some minority ethnic groups after adjustment for socioeconomic status, although rates remained significantly elevated compared with incidence in the white British group. Further, rates in the Caribbean do not appear to be elevated to the same extent as amongst their migrant counterparts [21–23]. Selten et al. [13] all-but excluded the possibility that selective migration of individuals predisposed to schizophrenia could account for the excess of disorder amongst Surinamese groups in the Netherlands, in a clever thought experiment which addressed this potential artefact.

Some artefactual explanations for raised rates of psychotic disorder in migrant groups and their descendants have continued to receive support, despite limited empirical evidence to support them. These include: the potential over-diagnosis of psychotic disorders in some minority ethnic groups by psychiatrists trained using a Western biomedical disease model [11], who may lack sufficient transcultural understanding of normal beliefs and behaviours which may be held by some immigrant populations; the over-diagnosis of schizophrenia in minority ethnic groups compared with other non-affective or affective disorders [11]; under-estimation of the minority ethnic population at-risk, particularly using early (pre-1991) census data in the UK which did not explicitly count the population by ethnicity, or 1991 census data which is known to have under-enumerated some minority ethnic groups [24], and; variation in other aspects of epidemiological enquiry (case finding, catchment definition, choice of baseline comparison group).

To further investigate some of these methodological issues in the context of raised incidence rates in minority ethnic groups, we sought to systematically review the epidemiological literature on the incidence of schizophrenia and other psychotic disorders in one major ethnic group in England, people of black Caribbean descent, in relation to rates in the background population. Using incidence rate data from a recently published systematic review in England [2], we had the opportunity to investigate whether the relative incidence of schizophrenia and other psychotic disorders among the black Caribbean population living in England had changed during 63 years of observation compared with the reference population, and any possible aetiological or artefactual reasons for this. While the excess risk of psychotic disorder in several ethnic minority groups creates a substantial public mental health burden in England, the present investigation focuses on the black Caribbean population because empirical evidence suggests that the excess risk in this population is particularly deleterious in public mental health terms [25, 26].

## Materials and methods

The current study is based on data from a recently published systematic review of the published, grey and unpublished literature on the incidence of schizophrenia and other psychoses in England, 1950–2009 [2], extended here until 31 December 2013. A brief overview of the original review methodology is provided, with particular details central to the current review afforded further expansion.

### Citation identification

A comprehensive list of search terms was developed and applied to several electronic databases (MEDLINE, Psych INFO, EMBASE, CINAHL, ASSIA and HMIC). This yielded 5,262 potential, unique citations, of which 83 met inclusion criteria following full paper review. We repeated this search strategy to identify suitable papers published between 2010 and 2013. This identified an additional 329 potential papers, of which two [27, 28] met our inclusion criteria:

- Time period: published 1950–2013.
- Extent: conducted wholly or partially in England.
- Scope: published, grey or unpublished literature.
- Contained original data on incident cases of non-organic, adult-onset psychotic disorder (15+ years) in people of black Caribbean descent and a comparator reference population.

We included the same diagnostic outcomes as in our previous review [2], which adopted a pragmatic approach to changing classification systems over time by synthesising data into the following broad categories by an experienced psychiatrist (PBJ): all psychotic disorders, non-affective psychotic disorders, schizophrenia (as a separate outcome), affective psychosis, bipolar disorder and psychotic depression (as separate outcomes) and substance-induced psychosis.

### Data extraction

We extracted information from all suitable papers to obtain incidence rate data, sample characteristics and other study-level “meta” variables. Rate-level variables included sample size (numerator), reported denominator, incidence rates (where available adjusted or standardised rates and rate ratios were chosen), standard errors and/or confidence intervals. Meta-level variables included the mid-point year of case ascertainment, study quality and urbanicity [2]. Study quality was assessed on a seven-point scale based on the presence of seven desirable methodological attributes (defined catchment area, accurate denominator estimation, population-based case finding, standardized research diagnoses, blinding to demographic factors, reporting of inclusion criteria, leakage study). Urbanicity of all study settings in the original report were ranked by several authors, with the mean rank taken to range from 1 (most urban) to 38 (least urban) (see [2]). We also classified studies according to the reference population used to compare rates in the black Caribbean groups, as either “UK-born”, “Remaining population” (only the black Caribbean or Caribbean-born groups excluded), “white” or “white British”. To inspect the possibility that differences in incidence rates could have been an artefact of case ascertainment, we classified each study as based either on hospital admissions, case register or first contact case finding. Finally we considered whether changes in the way the denominator was estimated by the Office for National Statistics [ONS] could have had a bearing on incidence rate ratios, classifying each study according to its Census (prior to 1991, 1991 census or 2001 census).

### Data synthesis

First, we provided a narrative synthesis of the results from studies included in this review, themed by diagnostic outcome. Second, where there was a sufficient number of studies (*N* ≥ 5) with adequate data, we estimated pooled incidence rates (IR) per 100,000 person-years (in the black Caribbean and reference group separately) and incidence rate ratios (IRR), and corresponding 95 % confidence intervals (95 % CI), via random effects meta-analysis. We assumed that separate studies represented a random sample from a population of studies which had a mean effect size (log IR, log IRR) about which individual study IR and IRR varied [29]. We reported the level of inconsistency amongst IR and IRR as estimated by the *I* ^2^-statistic [30], where values over 75 % were considered substantial. Third, we used random effects meta-regression to estimate the effects of meta-level explanatory variables on the outcome of interest (log IR, log IRR). We reported odds ratios [OR] for the change in IR or IRR associated with a one-unit change in the predictor of interest. Finally, to inspect possible small study (publication) bias, we constructed a funnel plot of each individual study’s IRR against its standard error (i.e., sample size) and performed Egger’s test for asymmetry in the resultant distribution, where *p* < 0.05 indicated possible bias. Analyses were performed in Stata 13 using user-developed commands for meta-analysis (metan, metareg, metabias, metafunnel).

## Results

### Study identification

**Fig. 1:**
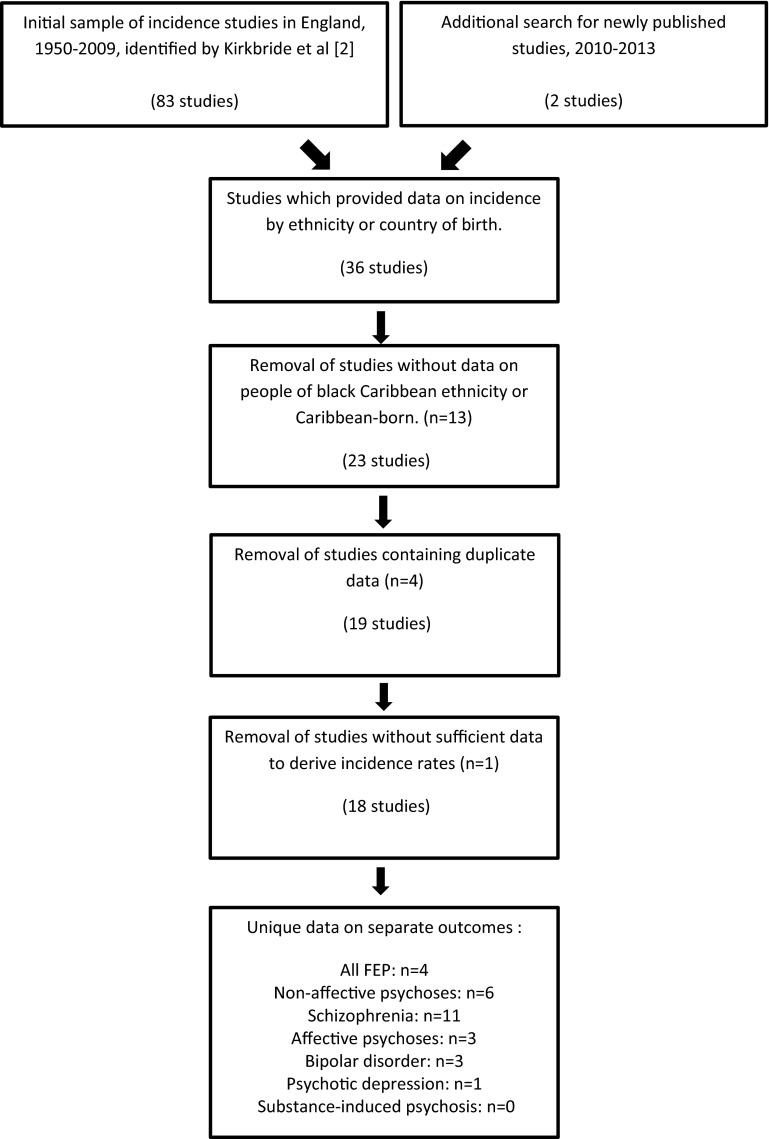
Flowchart of study identification

We identified 85 initial citations which provided incidence data on psychotic disorders in England between 1950 and 2013 (Fig. 1). Thirty-six studies provided data by ethnicity or country of birth, of which 25 pertained to incidence in people of black Caribbean origin or descent, of which two did not separate black Caribbean from black African groups [31, 32]. A further four studies duplicated data [28, 33–35] presented in other citations and one study [31] had insufficient data for inclusion (Fig. 1). Of the 18 remaining studies included in this review (Table 1), most (*n* = 11) provided incidence data in regard to schizophrenia [4, 10, 20, 36–43]. One of these [41] provided separate rates for four distinct periods (1965–1969, 1970–1974, 1975–1979, 1980–1984), which we treated independently to investigate changes over time. Furthermore, the AESOP study [4] provided incidence rates for three separate catchment areas (Southeast London, Nottingham and Bristol), which were also considered separately in this review.

**Table 1:** Included studies of all psychotic disorders (*N* = 18) and their main methodological aspects, ordered by case ascertainment year

References	Setting	Case ascertain-ment period	Census	Official Population estimate adjustments	Authors’ adjustments	Available diagnostic outcome data for black Caribbean and reference population
Hemsi [17]	Camberwell and Lambeth, London	1961	1961	No adjustment, Census data by country of birth and sex for catchment area	Crude first admission rates by sex and place of birth	Clinical diagnosis of schizophrenia. Classification system used not described
Rwegellera [45]	Camberwell, London	1965–1968	1966	Mid-term 1966 10 % sample Census	Adjustment for under-enumeration applied to all groups, officially estimated as 1.023	Hospital (clinical) diagnoses of non-affective and affective psychotic disorders, rated according to Schneiderian First Rank symptoms
Castle et al. [41] (schizophrenia) and van Os et al. [49] (mania)	Camberwell, London	1965–1969	1961	Population estimates for the intermediate years extrapolated	Direct standardisation to 1964 Census age structure Correction for missing cases for each cohort Correction 10 % under-enumeration for the Caribbean group	OPCRIT-based RDC “narrow schizophrenia”, “mania”
		1970–1974	1971			
		1975–1979	1981	Population estimates for the intermediate years extrapolated In the cohort 1980-1984: rates by born in the WI and rates by country of birth of head of household	Direct standardisation to 1964 Census age structure Correction for missing cases for each cohort Correction 10 % under-enumeration for the Caribbean group	OPCRIT-based RDC “narrow schizophrenia”, “mania”
		1980–1984				
Bebbington et al. [48]	Camberwell, London	1971–1977	1971	–	1971 Census +10 % sample for detailed estimates by age, sex and country of origin	ICD-9 Affective psychoses
Carpenter and Brockington [36]	Manchester	1973–1975	1971	Age distribution from 1971 census Number of migrants from 10 % Census of 1974	–	Clinical diagnosis of schizophrenia, affective psychoses (PSE)
Littlewood and Lipsedge [43]	Hackney, London	Unknown (18 months)	1971^a^	Not reported	Not reported. Crude first admission rates only available (no standard errors)	Clinical diagnosis of schizophrenia. No further details given.
Cochrane and Bal [42, 46]	England	1981	1981	–	Crude first admission rates by sex and place of birth	ICD-9 Non-affective psychoses [46] and schizophrenia [42]
Harrison et al. [10]	Nottingham	1984–1986	1981	1981 country of birth of head of household	–	ICD-9 Schizophrenia, mania
van Os et al. [37]	Camberwell, London	1988–1992	1991	1991 Census for previous and subsequent years Age-gender correction [24]	+ 20 % underreporting	RDC “narrow schizophrenia”
Bhugra et al. [38]	Ealing, South Southwark and East Lambeth, London	1991–1993	1991	Adjusted denominator for age, sex and ethnic group [24]	Narrow age bands standardization	CATEGO “Broad schizophrenia”
King et al. [39]	Haringey, Hackney and Enfield, London	1991–1992	1991		Age standardized +10 % underenumeration	ICD-9 Non-affective psychoses, schizophrenia
Harrison et al. [40]	Nottingham	1992–1994	1991	Adjusted denominator for age, sex and ethnic group [24]	Direct standardization for age	ICD-10 All first episode psychoses, schizophrenia
Fearon et al. [4]	Southeast London	1997–1999	2001	One number census methodology [78]	Indirect standardisation for age and sex	ICD-10 All first episode psychoses, non-affective psychoses, schizophrenia, bipolar disorder, psychotic depression
	Nottinghamshire					
	Bristol	1997				
Kirkbride/Coid et al. [20, 44]	East London	1996–1998 1998–2000	2001	One number census methodology [78]	Adjustment for age, sex and socio-economic status	DSM-IV All first episode psychoses, non-affective psychoses, schizophrenia, affective psychoses
Boydell et al. [27]	Camberwell, London	1998–2004	2001	One number census methodology [78]	No adjustment	RDC criteria for schizophrenia, schizoaffective disorder or mania

*WI* West Indies, *PSE* present state examination, *RDC* research diagnostic criteria, *ICD* international classification of diseases, *DSM* diagnostic and statistical manual, *OPCS* office for population and census statistics

^a^Not officially reported but inferred from reading the paper and, in particular, drawing on their original reference to the 1971 census [79]

### All clinically relevant psychotic disorders

Four studies, published between 1997 and 2013, provided data on the incidence of all clinically relevant psychotic disorders in the black Caribbean and reference population [4, 20, 27, 40]. One study was conducted as part of the AESOP study [4], one in East London [20], one from the Camberwell case register (South London) [27] and one in Nottingham [40]. Fearon et al. [4] observed an age-sex adjusted IRR of 6.7 (95 % CI 5.4–8.3) for all ICD-10 psychotic disorders in the black Caribbean group relative to the white British population (2001 denominator), using a population-based case finding approach. Rates were elevated for both men and women separately, and to a similar extent across all 5-year age bands between 16 and 64 years. In a methodologically-similar study in East London, based on DSM-IV criteria, age-sex adjusted IRR were also elevated in the black Caribbean group relative to the white British population, although were somewhat lower than reported elsewhere (IRR 4.0; 95 % CI 3.0–5.4). After additional adjustment for socioeconomic status, the IRR was further attenuated (IRR 2.7; 95 % CI 2.0–3.7). Boydell et al. [27] only reported crude IRR for RDC psychotic disorders (excluding psychotic depression) in the black Caribbean group relative to a general white group (2001 denominator); these were significantly elevated (IRR 8.1; 95 % CI 4.9–13.0). Finally, in the earliest study here, Harrison et al. [40] inspected rates of ICD-10 psychotic disorder in the black Caribbean group relative to the remaining population, as estimated from the 1991 Census denominator, corrected for under-enumeration. They reported age-sex standardised IRR in the black Caribbean group of 8.8 (95 % CI 6.0–12.9).

### Non-affective psychotic disorders

Six studies, published between 1977 and 2008, provided incidence data on all non-affective psychoses in the black Caribbean group relative to the reference population [4, 38, 39, 44–46]. All studies found elevated IRR in the black Caribbean group, which ranged from 1.8 in West London (relative to the white population) [38], through to 7.7 in a study of first generation Caribbean migrants compared with people born in England [45]. Fearon et al. [4] provided rates for Southeast London, Nottingham and Bristol separately, leading to eight unique study estimates of incidence, permitting meta-analysis. These results suggested the pooled IRR of non-affective psychosis in the black Caribbean group relative to the reference population was 5.0 (95 % CI 3.5–7.1; *I* ^2^ = 80.1; *p* < 0.001). Heterogeneity was driven by both the reference population incidence (pooled IR: 15 per 100,000 person-years; 95 % CI 11.1–21.7; *I* ^2^ = 95.3; *p* < 0.001) and rates in the Caribbean group (pooled IR 83.7; 95 % CI 63.1–111.0; *I* ^2^ = 81.1; *p* < 0.001). Meta-regression suggested the incidence of non-affective psychoses in the Caribbean group was lower when estimated based on hospitalised admissions rather than first contact study designs (OR 0.42; 95 % CI 0.18–0.95). This was not observed amongst the reference population (OR 0.66; 95 % CI 0.35–1.24). No other statistically significant variation by meta-level variables was observed. There was no evidence of small study bias from Egger’s test (*p* = 0.35).

### Schizophrenia

Available incidence data from eleven studies covered a 41-year period, with variation in case finding, reference population, denominator used, study quality and urbanicity (Table 2). Data predominantly came from settings in London (*n* = 7) [4, 17, 20, 37, 39, 41, 43] or Nottingham (*n* = 3) [4, 10, 40].

**Table 2:** Study- and meta-level data extracted from included studies of schizophrenia (*N* = 16), ordered by mid-point of case ascertainment

References	Ethnicity/reference	Cases (*n*)	Denom.	Incidence rates per 100,000 person-years	Rate ratios (95 % CI)	Midpoint year	Type of study	Urbanicity score	Study quality	Census denom. reliability
Hemsi [17]	Born WI	12	12,843	93.4	4.0 (3.3–4.8)	1961	Hospital admission	3	2	Before 1991
	UK born	47	199,465	23.6						
Castle et al. [41]	Born WI	9.3^f^	22,961	40.5	6.0 (2.9–11.9)	1967	Case register	5	5	Before 1991
	UK born	52.5^f^	776,987	6.8						
Castle et al. [41]	Born WI	23.8^f^	40,284	59.1	7.2 (4.4–11.7)	1972	Case register	5	5	Before 1991
	UK born	52.4^f^	642,025	8.2						
Carpenter and Brockington [36]	Born WI	23	6,906	111.0	5.6 (3.6–8.6)	1974	Hospital admission	21	2	Before 1991
	UK born	172	287,047	20.0						
Castle [41]	Born WI	20.6^f^	36,307	56.7	5.3 (3.2–8.7)	1977	Case register	5	5	Before 1991
	UK born	61.8^f^	578,639	10.7						
Cochrane and Bal [42]	Born WI	105.4^d^	285,193	37.0^e^	4.1 (2.2–7.7)	1981	Hospital admission	N/A	2	Before 1991
	UK born	2853.3^d^	31,303,340	9.0^e^						
Castle et al. [41]	Born WI	17.4^f^	45,144	38.1	1st gen vs UK-born: 3.2 (1.9–5.4) Black Caribbean vs. non black-Caribbean: 3.9 (2.4–6.2)	1982	Case register	5	5	Before 1991
	Black Caribbean	31.3^f^	76,660	39.8						
	UK born	59.3^f^	491,251	12.1						
Harrison et al. [10]	Black Caribbean	27	17,025	158.6	5.8 (3.6–9.1)	1985	First contact	25	4	Before 1991
	Remaining population	59	215,909	27.3						
van Os et al.[37]	Black Caribbean	22	63,953	34.4	4.4 (2.4–7.3) 3.1 (1.7–5.3)^b c^	1990	Hospital admission	5	4	1991
	White	30	384,615	7.8						
Bhugra et al. [38]	Black Caribbean	38	64,817	59.0 51.0^**a**^	2.0 1.8 (1.1–2.7)^a^	1992	First contact	5	6	1991
	White	38	124,767	30.0 29.0^**a**^						
King et al. [39]	Black Caribbean	9	14,973	53.0 48.0^**a**^	4.4 4.0 (1.7–9.1)^a^	1992	First contact	4	7	1991
	White	15	121,438	12.0						
Harrison et al. [40]	Black Caribbean	11	9,177	60.0 46.7^**a**^	10.0 8.2 (4.2–15.8)^a c^	1993	First contact	25	4	1991
	Remaining population	46	388,405	6.0 5.7^**a**^						
Fearon et al. [4] (London)	Black Caribbean	53	70,970	74.6	6.2 (4.2–9.4)	1998	First contact	8	6	2001
	White British	35	288,690	12.1						
Fearon et al. [4] (Nottingham)	Black Caribbean	8	16,260	49.2	7.5 (3.5–15.8)	1998	First contact	30	6	2001
	White British	47	713,716	6.6						
Fearon et al. [4] (Bristol)	Black Caribbean	2	3,392	58.9	9.1 (2.7–39.6)	1998	First contact	27	6	2001
	White British	15	230,674	6.5						
Kirkbride et al. [20]	Black Caribbean	48	65,070	73.7	4.2 (2.8–6.0)	1998	First contact	1	6	2001
	White British	61	345,078	17.7						

Standardised rates/rate ratios were used in pooled analyses, where available

*Denom*. denominator

^a^Standardised or adjusted for age

^b^Standardised or adjusted for age and sex

^c^Denotes use of standardized morbidity/incidence ratios instead of rate ratios

^d^Obtained directly from 1981 Census of Great Britain by present authors. Not originally reported

^e^Approximate estimates. Derived for both sexes by the present authors after estimating the 1981 denominator, given reported incidence rates for men and women separately. Not originally reported

^f^Correction for missing case notes made by Castle et al. [41]

### Narrative review

Of studies taking place in London, most were conducted in two inner-city boroughs, Lambeth and Southwark, home to a large proportion of Caribbean migrants and their descendants settling in England after the Second World War. Hemsi [17] first observed elevated first admission rates of schizophrenia in the Caribbean-born population relative to “persons born in the British Isles, including Eire” (pp. 96) in this community. His study preceded the Camberwell cumulative psychiatric case register, later established by John Wing [47], which enabled Castle et al. [33, 41] to conduct epidemiological studies of RDC schizophrenia incidence between 1964 and 1984. Substantially increased rates among patients born in the West Indies were observed compared with the reference UK-born population between 1965 and 1979 (see Castle et al. [41]; Table 2). This continued for the period 1980–1984, when elevated rates were observed for all black Caribbean groups, including British-born people of black Caribbean descent (RR 3.9; 95 % CI 2.5–6.0). Data from a later study in the same catchment area [37] provided a fifth time point (1988–1992) and was the first study to use the 1991 Census denominator. After standardisation for age and sex, and 20 % inflation of the black Caribbean denominator for census under-enumeration, excess rates remained present (RR 3.1; 95 % CI 2.0–7.3) compared with the white population. Finally, using 2001 census data and a population-based case-finding approach, Fearon et al. [4] observed elevated age-sex adjusted incidence rate ratios in black Caribbean groups relative to the white British population in all three centres of the AESOP study.

Elsewhere in London, elevated rates of schizophrenia in the black Caribbean population have also been observed; Littlewood and Lipsedge [43] observed higher rates in Caribbean-born migrants in East London, albeit in a study lacking the epidemiological rigour of contemporary research (Table 2). However, this excess was confirmed in the same catchment, two decades later, by Kirkbride et al. [20], which persisted after adjustment for age, sex and socioeconomic status. In two studies conducted in the 1990s in West and North London [38, 39], respectively, elevated rates of schizophrenia were also observed in the black Caribbean population relative to the white population estimated from the 1991 census.

Three relevant studies, including the aforementioned AESOP study, were conducted in Nottingham, between 1984 and 1999 [4, 10, 40]; all three employed a first contact design, but were based on increasingly precise population at-risk estimates from three decennial censuses. Harrison et al. [10] observed schizophrenia rate ratios in excess of 5.8 for the black Caribbean group compared with the rate in the general population using 1981 census data. A second study [40] reported even higher rates when compared against the narrower reference “white” population group (RR 8.1; 95 % CI 4.2–15.8) from the 1991 census, a finding which persisted in further data from AESOP study using a white British reference from the 2001 census (RR 7.5; 95 % CI 3.5–15.8). The incidence of schizophrenia in Caribbean-born migrants was also elevated in data from Manchester [36] and the Mental Health Enquiry of England [42], relative to the English-born population, as well as amongst the black Caribbean group in the Bristol centre of the AESOP study (Table 2) [4].

### Meta-analyses and meta-regression of schizophrenia incidence rates and rate ratios

**Fig. 2:**
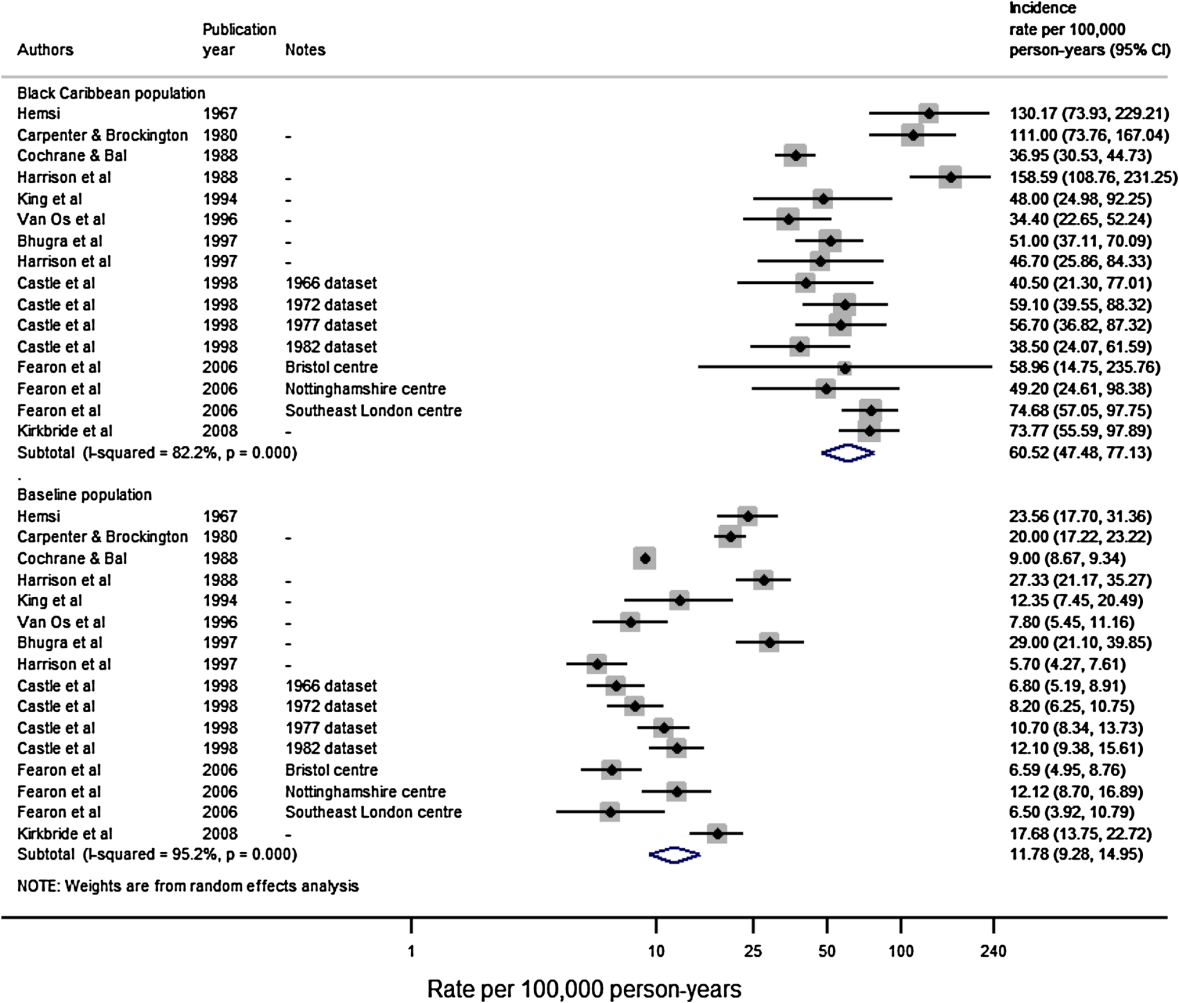
Pooled incidence of schizophrenia in the black Caribbean and reference population in England

**Fig. 3:**
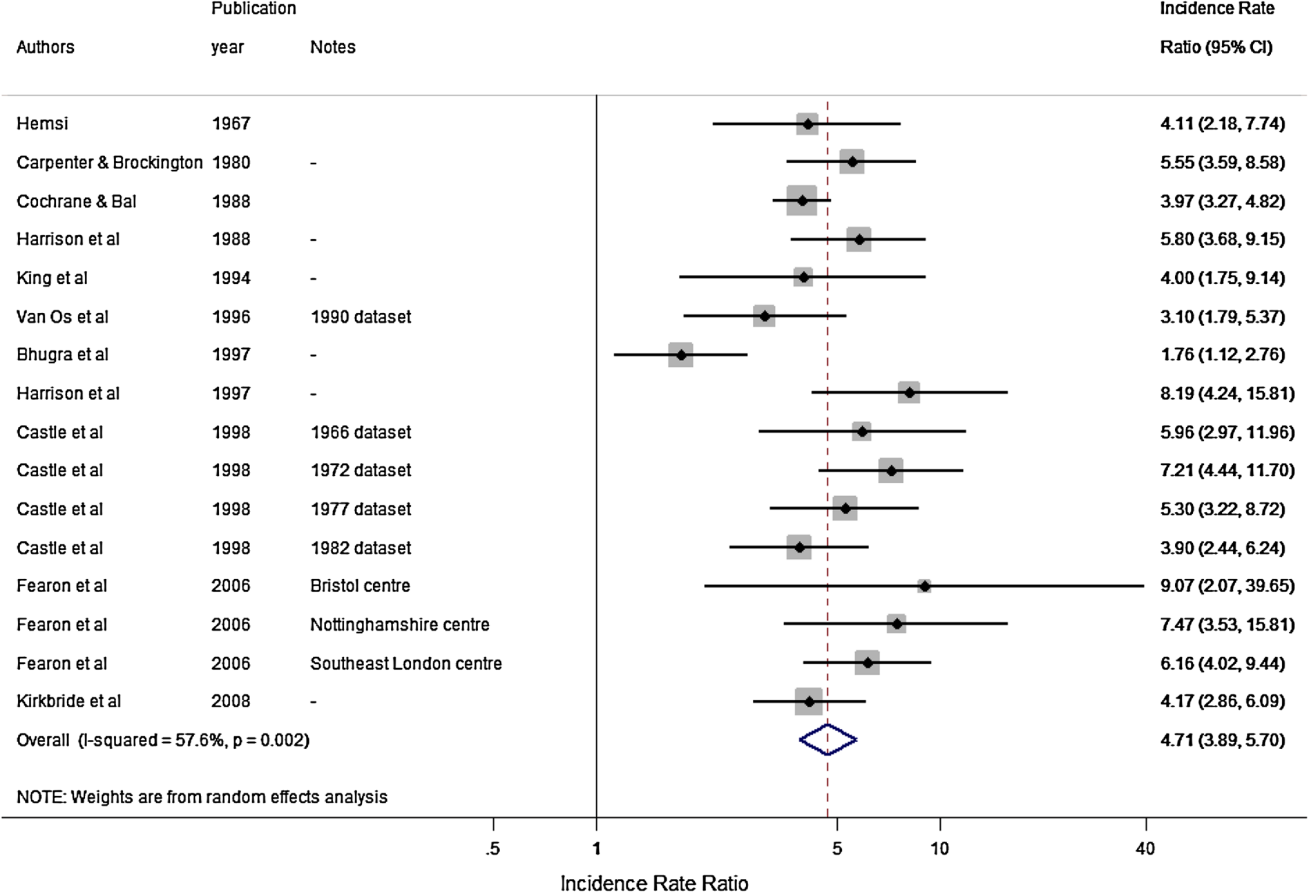
Pooled incidence rate ratios of schizophrenia in the black Caribbean group compared with the reference population in England

The pooled IR of schizophrenia from the eleven studies conducted over 16 unique settings/time points was 11.8 cases per 100,000 person-years in the reference population (95 % CI 9.3–14.9; *I* ^2^ = 95.2; *p* < 0.001) and 60.5 (95 % CI 47.5–77.1; *I* ^2^ = 82.2 %; *p* < 0.001) in the black Caribbean group (Fig. 2). Heterogeneity in both samples was high. The pooled IRR across these studies was 4.7 (95 % CI 3.9–5.7; *I* ^2^ = 57.6 %; *p* = 0.002) (Fig. 3).

We found no initial evidence from meta-regressions to suggest IR in either the black Caribbean or reference population varied significantly over time, by urbanicity, study quality, denominator population used, reference population used or case finding approach (Table 3). However, there was a trend to suggest that IRR reported in more urban areas were smaller than those from more rural settings (OR for change in IRR associated with a one rank increase in urbanicity: 0.98; 95 % CI 0.96, 1.00; *p* = 0.06). We also observed that studies using the white population as a reference tended to report lower IRR than those using a narrower “white British” definition (OR 0.46; 95 % CI 0.27, 0.77; *p* < 0.01). A funnel plot of IRR plotted against their standard error (sample size) revealed little evidence of publication bias (Online Figure 1), confirmed by Egger’s test for asymmetry (*p* = 0.21).

**Table 3:** Summary of meta-regression effects for incidence rates and rate ratios for schizophrenia, by meta-level variables

Meta-level variable	Black Caribbean incidence		Reference population incidence		Incidence rate ratios	
	OR	95 % CI	OR	95 % CI	OR	95 % CI
Time (years)	0.99	0.97, 1.02	0.99	0.97, 1.02	1.00	0.98, 1.02
Urbanicity rank^a^	0.99	0.96, 1.01	1.01	0.98, 1.04	0.98	0.96, 1.00^
Study quality^b^	0.93	0.77, 1.12	0.94	0.77, 1.14	0.99	0.86, 1.15
Denominator reliability						
2001 census	1		1		1	
1991 census	0.67	0.30, 1.52	1.13	0.47, 2.71	0.59	0.32, 1.08
Pre–1991 census	1.02	0.51, 2.07	1.31	0.62, 2.78	0.89	0.54, 1.48
Reference population						
White British	1		1		1	
White	0.66	0.30, 1.44	1.43	0.53, 3.89	0.46	0.27, 0.77*
UK-born, British	0.89	0.46, 1.70	1.18	0.53, 2.64	0.88	0.59, 1.32
Remaining population	1.40	0.57, 3.39	1.26	0.42, 3.82	1.21	0.67, 2.16
Case finding method						
First contact	1		1		1	
Hospital admission	1.09	0.54, 2.18	1.23	0.56, 2.68	0.90	0.52, 1.54
Case register	1.09	0.56, 2.10	1.04	0.50, 2.17	1.19	0.70, 2.02

*OR* odds ratio, *95* *% CI* 95 % confidence interval

* *p* < 0.01 ^ *p* = 0.06

^a^Urbanicity rank varied across settings from 1 (most urban) to 30 (most rural) out of 38 possible rankings, based on the original 83 citations identified in Kirkbride et al. [2]. Here, the ranking is reversed, so odds ratios below one denote the reduction in incidence or rate ratio associated with a one rank increase in urbanicity. Rates from Cochrane and Bal [42] are not included in this analysis as this study was based on data for the whole of England

^b^Observed study quality ranged from 2 to 7 on a theoretical scale from 0 to 7, as originally defined by Kirkbride et al. [2]

### Affective psychotic disorders

Three studies reported incidence data of all affective psychotic disorders in the black Caribbean and reference populations living in England [20, 36, 48]. The two earlier studies found no evidence to suggest that first admission rates of affective psychoses were elevated in Caribbean-born migrants relative to the British-born population in samples from Manchester [36] and South London [48]. By contrast, the most recent population-based incidence study of affective psychoses in these populations [20] observed elevated rates of affective psychoses in the black Caribbean group relative to the white British population, which persisted despite adjustment for age, sex and socioeconomic status (IRR 2.4; 95 % CI 1.3–4.3).

This latter finding is supported by data from the AESOP study [4], which observed that IRR for both bipolar disorder (IRR 8.0; 95 % CI 4.3–14.8) and psychotic depression (IRR 3.1; 95 % CI 1.5–6.1) were elevated in the black Caribbean group relative to the white British population, after adjustment for age and sex. Harrison et al. [10] also reported elevated crude first admission rates of “mania” in black Caribbean groups (IR 38.4 per 100,000) in Nottingham relative to the general population (IR 3.2 per 100,000), although the sample of Caribbean participants was small (*n* = 4). In Camberwell, van Os et al. [49] reported statistically-significant elevated crude rates of mania and schiozomania at every five year time point between 1965 and 1984 using case register data in the Caribbean-born population compared with their UK-born counterparts.

## Discussion

### Principal findings

Our systematic review has sought to identify all literature on the incidence of psychotic disorders in the black Caribbean community in England published over a 63 year period. Of eighteen studies which met inclusion, 16 identified a statistically significant elevation in risk of psychotic disorder in the Caribbean group relative to the reference population under study; only two early studies of affective psychoses did not [36, 48]. Elevated rates were not, however, limited to schizophrenia, for which the pooled IRR in the black Caribbean group was almost five times greater than the reference population. Studies varied in methodological quality. However, where meta-regression was possible (for non-affective psychoses and schizophrenia separately) we found little evidence to suggest that methodological features of study design or observation were associated with changes in the reported IR or IRR between black Caribbean and reference groups. One notable exception to this was with respect to the definition of reference population; studies which used a white (any background) population reported significantly lower IRR for schizophrenia than those using the narrower “white British” reference. Given some reports of elevated rates of psychotic disorders in some non-British white minority groups in England [4, 20], use of a broad white reference group is likely to have biased IRR in the black Caribbean population toward the null. Finally, we observed a trend towards smaller IRRs for schizophrenia in the black Caribbean population in studies conducted in more urban settings.

### Artefactual considerations

We used a comprehensive search and selection strategy of the published, grey (via HMIC) and unpublished literature over seven decades, based on a validated and reliable methodology [2] to minimize missed studies. While conservative in the presence of substantial heterogeneity, Egger’s test for small study bias did not indicate any evidence of substantive publication bias for either non-affective psychoses or schizophrenia as diagnostic categories. We identified one study by Bhavsar et al. [50] published after the end-point of our review (2014), which also confirmed an elevated incidence of RDC schizophrenia in the black Caribbean population, aged 16–35 years, in South London identified via an early intervention psychosis service between 2000 and 2007 (adjusted IRR 12.0; 95 % CI 1.7–86.8).

To minimize problems surrounding heterogeneity we included a strong narrative synthesis of available data from individual studies. Nevertheless, the methodologies of individual studies were heterogeneous and pooled effect sizes should be interpreted in line with corresponding *I* ^2^-statistics. These were smaller for pooled rates of non-affective psychoses and schizophrenia in the black Caribbean group than the baseline group, perhaps unsurprising given the particularly varied choice of reference definitions used over time; meta-regression of schizophrenia studies confirmed that use of a white reference group led to smaller IRR for the black Caribbean group than use of a narrower “white British” group. Heterogeneity remained high in the black Caribbean group, and we recognize that we did not have sufficient resolution from individual studies (or from UK census denominator data) to distinguish between groups from different Caribbean islands, who may have different socioeconomic and cultural histories. We treated data from three separate centres of the AESOP study as independent samples, which might have artificially lowered heterogeneity in some results.

We chose a pragmatic approach to diagnosis of psychotic disorders [2], given changing classifications over time and between studies. This approach has construct validity with overall pooled IRs in the total population (see [2]) in-line with those from international meta-analyses [51]. Our results indicate that people of black Caribbean ethnicity in England had elevated incidence rates of all psychotic disorders studied (except substance-induced psychoses where no empirical data was available), making over-diagnosis of schizophrenia in preference to other psychotic disorders unlikely. This is distinct from the possibility that psychiatrists misdiagnose normal cultural beliefs, behaviours and mores as psychotic. Although we were unable to investigate that issue here, overall, there is little evidence to support such a systematic bias [52, 53]. There is good evidence that minority ethnic groups, including the black Caribbean group, have more complex pathways to care [54–56] and may receive worse mental health care [57]. These important issues are separate to the elevated rates of psychotic disorders seen in these groups.

Given the small number of data points available for meta-analysis, our meta-regressions may have been underpowered to detect variation by methodological facets of study design. For this reason, no attempt was made to perform multivariable meta-regressions.

Our findings suggested that improved study quality over time (correlation for studies pertaining to schizophrenia: 0.62; *p* = 0.01) had little overall impact on estimates of incidence or IRR. A similar observation was made by Bourque et al. [1] in their recent meta-analysis of the international literature on incidence rates in first- and second-generation migrants. One possibility is that the combination of a movement towards population-based case-finding approaches over time (correlation 0.73; *p* < 0.01) with more precise denominator estimates in minority ethnic groups led to overall homeostasis in terms of estimated IR and IRR, despite advances in study methodology. There was no evidence that IRR for non-affective psychoses or schizophrenia had changed systematically over several decades of observed data. The weak association between smaller IRR for schizophrenia and urbanicity may have been a chance finding. Related to this, sampling variation may have explained this result, since studies from more urban areas tended to be larger, and may therefore have been able to more accurately estimate IRR. Nevertheless, future observational studies could examine whether the excess risk of psychotic disorders in the black Caribbean population was less marked in more urban communities within individual studies.

### Aetiological considerations

Although some studies only presented unadjusted rates of psychotic disorder in black Caribbean populations in England (i.e. [17, 27]), most studies were able to control for potential confounding by age and sex. These studies demonstrated that elevated rates of all disorders, including mania and psychotic depression [4], persisted after this adjustment. Only three studies [4, 20, 38] of schizophrenia provided data by gender in the black Caribbean group; rates were increased for both black Caribbean men and women, with point estimates for IRR slightly higher amongst black Caribbean women than men in two of these studies [4, 20]. Only one study to date has been able to control for individual-level socioeconomic status [20]. In that study incidence rates remained two- to four- times higher in the black Caribbean group (compared with white British rates) for schizophrenia, other non-affective psychoses and the affective psychoses after additional adjustment for socioeconomic status. Other European studies have reported similar findings [58, 59], suggesting that the remaining excess risk in minority ethnic groups may be due to exposure to other contextual or environmental factors. Cannabis use may be one such factor, but to our knowledge no epidemiological incidence study in England or elsewhere has been able to control for cannabis use as a potential confounder of the association between psychosis and minority status. Some evidence from the UK suggests cannabis use is not more frequently reported amongst the black Caribbean population than their white British counterparts [60–62], nor in clinical samples [63], but explicit epidemiological data is required to test this hypothesis. Wider societal exposures, including social deprivation [50, 64–66], population density [65, 66] and inequality [65] are associated with total psychosis incidence in the population, but do not explain the excess risk in black and minority ethnic groups. As per the original studies in our review, we were not able to control for other potential confounders, including education, family history of psychiatric disorders or paternal age.

Cumulative social disadvantage [67] and adverse life events in childhood [68] have been associated with a similar increase in schizophrenia risk across several ethnic groups, but such exposures appear to be more prevalent amongst some minority ethnic populations, and particularly for black Caribbean and African communities in England. It is possible that the greater impact of these events may partially explain excess risk in these groups, although it is difficult to quantify the magnitude of potential confounding effects in incidence-based studies, given the difficulty of obtaining corresponding exposure data from routine denominator sources. Strong social cohesion may help to buffer the effect of exposure to some of these experiences, as evidenced by studies which show that the elevated incidence of non-affective psychotic disorders is attenuated for minority ethnic groups, when people live in closer proximity to others from their own ethnicity [65, 69, 70].

Four studies [50, 64, 70, 71] have investigated the role of ethnic density on incidence rates of non-affective psychotic disorders in South London, home to a large proportion of people from black Caribbean backgrounds. Two of these studies observed evidence of the ethnic density effect for all minority ethnic group members in relation to the overall proportion of the total minority ethnic population at the neighbourhood level [70, 71]. A third study [50] did not observe this effect, but a fourth found specific evidence for the ethnic density effect in people from black ethnic backgrounds [64]. Contextual ethnicity effects may also operate differentially by ethnic group. In East London, for example, Kirkbride et al. [65] observed evidence for the ethnic density effect amongst black African groups, but not in the black Caribbean population. There was evidence, however, that greater segregation of the black Caribbean group from other ethnic groups was associated with higher rates of disorder in this population. One possibility, therefore, is that both ethnic density and segregation are markers of social cohesion amongst minority ethnic groups, which buffer against exposure to social adversities (or their potentially deleterious consequences) [72], including discrimination [73], low socioeconomic status [20] or other social adversities [67, 68]. In our review we observed a weak association between greater urbanicity and lower IRR of schizophrenia in the black Caribbean group, potentially consistent with these effects, if people of black Caribbean origin in more rural populations faced greater exposure to social adversity.

Very few studies in England have examined incidence rates of schizophrenia in first- versus second- or later-generation black Caribbean groups separately. It is reasonable to assume that earlier studies (i.e., 1950–1980s) were probably based predominantly on first generation migrants. Later studies will have contained an increasing proportion of second- and later-generation migrants for whom elevated rates of psychotic disorder have continued to be observed [4, 20, 37, 38, 40]. One study [44] also observed elevated rates of non-affective psychotic disorder in people of mixed white and black Caribbean ethnicity.

We also observed higher rates of affective psychotic disorders among Caribbean migrants and their descendants in England. In general, affective disorders appear to be less influenced by the wider social environment (i.e., deprivation, inequality, fragmentation) than their non-affective counterparts [65, 74], suggesting that social stressors may also only play a partial role in explaining the excess risk of bipolar disorders observed in minority ethnic populations. Biological factors may also be relevant, including early life infections, malnutrition and the role of neuroinflammation. One untested hypothesis is that more severe social adversities (abuse, bullying, discrimination, migration, aberrant separation from a parent, parental death) play a role in the excess risk of *any* psychotic disorder, since severity is sufficient to overwhelm normal stress responses. However, more distal environmental factors (social deprivation, fragmentation, urbanicity) may require additional insults deficits, such as neurocognitive impairment (arising from, for example, altered early life neurodevelopment or genetic predisposition [75, 76]) to adversely influence psychosis risk; we know that people experiencing non-affective psychotic disorders tend to experience premorbid cognitive declines, not consistently seen amongst people with bipolar disorders [77].

In summary, our review has confirmed higher rates of all major psychotic disorders amongst black Caribbean migrants and their descendants in England over more than 60 years of observation. The different study methods and denominator calculations used over time and across studies did not explain the higher rates found among this population. Moreover this risk remained present despite improvements to study methodologies over time, and may have an inverse relationship with urbanicity. Many hypotheses have been put forward to explain raised rates of schizophrenia and other psychoses in black Caribbean and other ethnic minority and immigrant populations (for example, see [11]). There is now reasonable evidence against some of these, including higher rates in the country of origin [21–23], misdiagnosis (see above) or selective migration [13]. We suggest that the continued excess incidence of non-affective and affective psychotic disorders in black Caribbean and other minority ethnic groups will be most parsimoniously explained by increased exposure to social and economic disadvantage in the post-migratory environment.

## Electronic supplementary material

Below is the link to the electronic supplementary material.

Supplementary material 1 (DOCX 64 kb)
