# Supplementary material for: Schizophrenia and other psychotic disorders in Caribbean-born migrants and their descendants in England: systematic review and meta-analysis of incidence rates, 1950–2013
Source: Soc Psychiatry Psychiatr Epidemiol. 2015 Feb 7;50(7):1039–55. doi: 10.1007/s00127-015-1021-6 (PMC4464051; doi:10.1007/s00127-015-1021-6)
Supplement: Supplementary file 1 — Supplementary material 1 (DOCX 64 kb) [file 127_2015_1021_MOESM1_ESM.docx]

**Online Figure 1: Funnel plot of incidence rate ratios of schizophrenia in the black Caribbean versus reference group in England from included studies, 1950-2013**

**
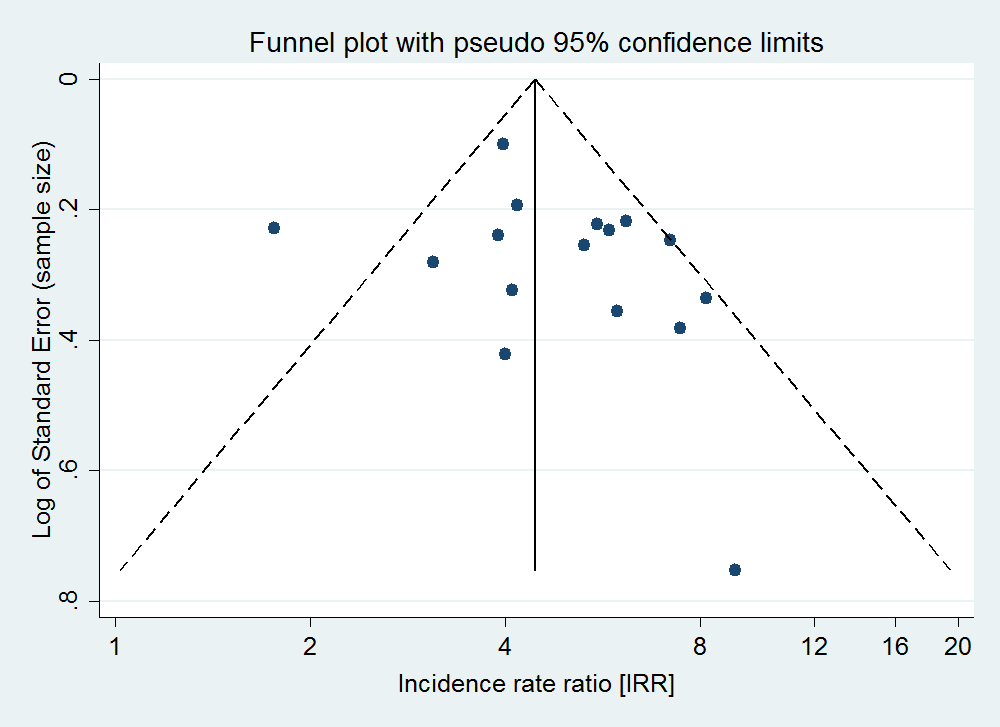
**

Figure caption: A funnel plot for schizophrenia did not suggest any evidence of small study (publication) bias, confirmed by an Egger’s test (p=0.21).
